# LncRNA-MSC-AS1 inhibits the ovarian cancer progression by targeting miR-425-5p

**DOI:** 10.1186/s13048-021-00857-2

**Published:** 2021-08-28

**Authors:** Yinling Zhao, Donglan Yuan, Dandan Zhu, Tianhui Xu, Aihua Huang, Li Jiang, Chiwen Liu, Hua Qian, Xinhua Bu

**Affiliations:** Department of Obstetrics and Gynaecology, Taizhou People’s Hospital, 399 Hailing South Road, Taizhou, 225300 Jiangsu China

**Keywords:** LncRNA MSC-AS1, MicroRNA-425-5p, Proliferation, Apoptosis, Ovarian cancer

## Abstract

**Background:**

Long non-coding RNAs (lncRNAs) and microRNAs (miRNAs) were reported to be aberrantly expressed and related to the pathogenesis of ovarian cancer. However, the role and regulatory mechanism of MSC-AS1 in ovarian cancer has yet to be fully elucidated.

**Methods:**

Expression of lncRNA MSC-AS1 (MSC-AS1) and microRNA-425-5p (miR-425-5p) in the ovarian cancer tissue samples and cell lines was examined by quantitative real-time polymerase chain reaction (qRT-PCR). The functions of MSC-AS1 on ovarian cancer cell proliferation, cell cycle and apoptosis were determined using MTT, colony formation and flow cytometry analyses. The protein expression levels were evaluated using western blot assay. The targeting relationship MSC-AS1 and miR-425-5p was verified via dual-luciferase reporter assay.

**Results:**

MSC-AS1 expression level was lowly expressed, while miR-425-5p level was highly in ovarian cancer tissues and cells. Elevation of MSC-AS1 has the ability to significantly inhibit cell proliferation and facilitate cell apoptosis in SKOV3 and A2780 cells. Moreover, MSC-AS1 targeted and negatively modulated miR-425-5p. MiR-425-5p up-regulation has been proved to partially reverse the tumor suppressive function of MSC-AS1 overexpression

**Conclusion:**

MSC-AS1 sponged miR-425-5p to inhibit the ovarian cancer progression. These findings may provide a promising therapeutic target for the treatment of ovarian cancer.

## Introduction

Ovarian cancer is one kind of the most common gynecologic malignancy worldwide, which has high mortality [1, 2]. The patients was diagnose at advanced stage and the low diagnostic accuracy in the early stage [3]. Although the rise of great advancements in therapeutic strategies for ovarian cancer patients, including surgery, radiotherapy and chemotherapy, the five-year survival rate of ovarian cancer is still unsatisfied [4–6]. Nonetheless, molecules that are suitable for targeted therapies are still limited. Consequently, to explore the molecular mechanisms and to find potential targets were urgently needed for ovarian cancer diagnosis and therapy to improve the survival quality of ovarian cancer patients.

Long non-coding RNAs (lncRNAs) are a category of non-coding transcripts with longer than 200 nucleotides, which have attached more and more attention of researchers. A growing number of studies have disclosed that lncRNAs may play a tumorigenic or tumor-suppressive role in tumor carcinogenesis, ovarian cancer contained. For instance, lncRNA GAS5 regulates liver cancer proliferation and drug resistance by decreasing PTEN expression [7]. Wu et al. pointed out that knockdown of lncRNA PVT1 inhibits prostate cancer progression by regulating miR-15a-5p /KIF23 axis [8]. Interestingly, lncRNA H19 promotes cell proliferation and metastasis by inhibiting miR-200a expression in lung cancer [9].

LncRNA MSC-AS1 is newly discovered in recent years. Studies have found that contributes to many tumor progression. For example, MSC-AS1 might promote the osteogenic differentiation of BMSCs [10]. In addition, lncRNA MSC-AS1 aggravated NPC progression by sponging miR-524-5p to increase NR4A2 expression [11]. Hu et al reported that MSC-AS1 regulates KIRC cell proliferation and migration via miR-3924/WNT5A/β-catenin axis [12]. Based on TCGA data, a significant low expression of MSC-AS1 was observed in ovarian cancer tissues. However, no study was performed on the role of MSC-AS1 in ovarian cancer. Thus, its function in ovarian cancer requires further investigation. For this study, we designed and conducted experiments to clarify the function of the MSC-AS1 in ovarian cancer and its underlying mechanisms. Elucidating the function and potential mechanisms of lncRNAs and miRNAs in ovarian cancer may provide markers for ovarian cancer diagnosis and therapy.

MicroRNAs (miRNAs) are a family of small non-coding RNAs with the length of 18-22 nucleotides. These short miRNAs bind to the 3’ untranslated region (3’UTR) of their target transcripts. Similar to lncRNA, numerous studies have reported the abnormal expression of miRNAs participate in the development of tumors, such as proliferation, invasion, differentiation and apoptosis. For instance, previous studies depict that miR-23a promotes colorectal cancer cell migration and proliferation by targeting at MARK1 [13]. Furthermore, microRNA-21 could promotes cell proliferation and metastasis by targeting LZTFL1 in breast cancer [14]. Mai et al displayed that miR-18a promotes cancer progression through SMG1 suppression in nasopharyngeal carcinoma [15]. In breast cancer, miR-425-5p is associated with poor prognosis, and promotes cell proliferation and migration via PTEN [16]. Moreover, miR-425-5p was upregulated in hepatocellular carcinoma tissues and cell lines, and promotes cell proliferation, migration and invasion by directly targeting FOXD3 [17]. Liu et al suggested that miR-425-5p suppresses tumorigenesis and DDP resistance by targeting GSK3beta and inactivating the Wnt/beta-catenin signaling pathway in prostate cancer [18]. More importantly, a previous study indicated that miR-425-5p is associated with renal cell carcinoma cell migration, proliferation and apoptosis [19]. In our current study, bioinformatics analysis and RT-qPCR examination found the highest binding potential of miRNA with MSC-AS1. MiR-425-5p has been reported to dysregulate in various cancers, including breast cancer [16], osteosarcoma [20], hepatocellular carcinoma [17] and colorectal cancer [21]. However, its interaction with MSC-AS1 has never been explored in ovarian cancer. In the present study, we demonstrated the interaction between MSC-AS1 and miR-425-5p through mechanism investigation

The present study intended to probe the expression pattern and potential roles of MSC-AS1 in ovarian cancer by gain-of-function assays. The results indicated that MSC-AS1 was down-regulated in ovarian cancer tissues and cells, and MSC-AS1 over-expression could inhibit cell proliferation and induce apoptosis by targeting miR-425-5p in ovarian cancer. Our findings may provide strong evidence in the molecular mechanism through which MSC-AS1 regulates the ovarian cancer progression.

## Materials and methods

### Tissues collection

Total 20 pairs of ovarian tumor tissues and normal tissues were collected from patients who underwent surgical resection at Taizhou People’s Hospital between June 2018 and April 2019. All patients for participation provided informed consent and ethical approval was obtained from the ethics committee of Taizhou People’s Hospital (Number: KY201804701). Tissues were stored immediately at -80°C.

### Cell culture

Ovarian cancer cell lines (OVCAR3, A2780, SKOV3) and human ovarian epithelial cell line (IOSE80) were purchased from the Shanghai Bank of Cells, Chinese Academy of Science (Shanghai, China). The cells were maintained in RPMI-1640 (Hyclone, USA) supplemented with 10% fetal bovine serum (FBS; Gibco) in a 37°C incubator with 5% of CO_2_.

### Cell transfection

MSC-AS1 overexpression vector (pcDNA 3.1 MSC-AS1), empty vector (pcDNA3.1), miR-425-5p mimic and mimic negative control (NC) were synthesized by GenePharma (Shanghai, China) which were employed to induce MSC-AS1 or miR-425-5p overexpression. Cell transfection was conducted at concentration of 50 nM utilizing Lipofectamine 2000 (Invitrogen; Thermo Fisher Scientific, Inc.) according with the manufacturer’s instruction. The samples were collected after 48 h transfection for further analysis.

### Quantitative Real-Time Polymerase Chain Reaction (qRT-PCR)

Total RNA was extracted from cells utilizing TRIzol reagent (Invitrogen). RNA was reverse transcribed to cDNA was acquired through Prime Script TM RT reagent kit (Takara, Dalian, China) and RNA quantification was assessed by Power SYBR Green (Takara, Dalian China) on ABI PRISM 7900 Real-time PCR system (Applied Biosystems). GAPDH and U6 were validated as the normalization. Gene relative expression level was calculated by the 2^−ΔΔCt^ method. The reaction conditions were as follows: 94°C 3min, 94°C 45s, 57°C 45s, 72°C 45s, a total of 30 cycle, and finally extended at 72°C for 10min. The specific primers were as follows: MSC-AS1 forward 5′-GCCAGTCAGAAAATGAGGAAC-3′ and reverse 5′-CCAGTTGGGTGAACAGGAC-3′; miR-425-5p forward 5′-GGGGAGTTAGGATTAGGTC-3′ and reverse 5′-TGCGTGTCGTGGAGTC-3′; GAPDH forward 5′-CTCAGACACCATGGGGAAGGTGA-3′ and reverse 5′-ATGATCTTGAGGCTGTTGTCATA-3′.

### Western blot analysis

Total protein in cells was extracted by RIPA solution (GenePharma) and the protein concentration was estimated through BCA Protein Assay Kit (Beyotime). Equal amounts protein samples were loaded on each lane and separated by 10% SDS-PAGE and then transferred onto a PVDF membrane (Millipore, Billerica, MA, USA). Next, the membrane were cultivated with primary antibodies against PCNA (ab92552, 1:1,000 dilution, abcam), Ki-67 (ab245113, 1:10,000 dilution, abcam), Bax (ab81083, 1:1,000 dilution, abcam), Caspase-3 (218161, 1:1000 dilution, abcam) and GAPDH (ab181603, 1:1,000 dilution, abcam) at 4°C overnight. The membrane was washed with PBST for three times. Then the membrane was incubated with the horseradish peroxidase (HRP)-conjugated secondary antibodies (ab6721, 1:1,000 dilution, abcam). The bands were visualized using a chemiluminescence detection kit (Beyotime).

### MTT assay

Cell (1 × 10^5^) viability was determined by MTT Kit (Beyotime, Shanghai, China). The cells were seeded in 96-well plates and incubated for 24, 48, or 72 h. 10 μL MTT was added with for 4 h. Then, 100 μL DMSO was added to each well and incubated with for 2 h. The optical density (OD) value was measured at 490nm wavelength and each experiment was repeated for three times.

### Colony formation assay

Cells (1 × 10^5^) were plated onto 6-well plates and maintained in complete culture medium for another 14 days. Then the cells were washed with PBS and fixed in 4% paraformaldehyde for 1 h, following stained with 0.1% crystal violet solution for 30 min at room temperature. Images of cells were captured under a light microscope and the number of clones was manually calculated in three randomly selected fields.

### 5-ethynyl-20-deoxyuridine (EdU) analysis

Cell proliferation was also determined using the EdU assay kit (Thermo Fisher Scientific) according to the manufacturer’s instructions. Cells (1 × 10^5^) were maintained in 6-well plates. After 48 h, 100 μL EdU was added for 2 h. Cells were incubated with 4% formaldehyde, followed by 0.3% Triton X-100 for 10 min. EdU-positive cells were analyzed using fluorescence microscopy (Olympus, Tokyo, Japan) at the 20× objective.

### Flow cytometry analysis

Cells were harvested and fixed for about 8 h fixation. Then, the cells were incubated with RNase (50 μg/ml) and propidium iodide (PI) (50 μg/ml, Thermo Fisher, Waltham, MA, USA) for 30 min. Then, the cell cycle was measured by Flow Cytometry System (BD Accuri C6) and the relative ratios of G0/G1, S and G2/M phases were analyzed by FlowJo VX software (BD Biosciences, Franklin Lakes, NJ, USA).

### Terminal deoxynucleotidyl transferase (TdT)-mediated dUTP nick end labeling (TUNEL) assay

TUNEL assay was performed to detect the apoptosis of transfected cells using One Step TUNEL Apoptosis Assay Kit (Beyotime Biotechnology Co., Ltd; China) following the manufacturer's instructions. Briefly, a total of 5 × 10^3^ cells were seeded into the 96-well plates for 48 h culture. Then, the cells were fixed with 4% paraformaldehyde for 30 min at room temperature. Subsequent to washing by PBS twice, 0.1% TritonX-100 was added to permeabilize the cell membrane. The cells were successively treated with TUNEL reaction mixture, converter-POD and DAPI substrate. The cell images were then obtained under the fluorescence microscope (Olympus, Tokyo, Japan).

### Dual luciferase reporter assay

To validate the binding between MSC-AS1 and miR-425-5p, the wild type and mutant type of MSC-AS1 3′UTR (MSC-AS1-WT/MUT) were cloned into pmirGLO vector (Promega Corporation), and the cells were seeded into 24-well plates until reaching 60% confluence. Each well was co-transfected with luciferase reporter plasmids (0.5 μg) and miR-425-5p mimic or NC mimic (100 nM) into cells using Lipofectamine 2000 (Invitrogen) according to the manufacturer’s protocol. After transfection for 48 h, the luciferase activity was determined using dual-luciferase reporter assay system (Promega, Madison, WI, USA). The activity levels were normalized to those corresponding to the Renilla signals.

### Statistical analysis

All experiments were performed in triplicate and data were presented as mean ± SD. GraphPad Prism (version 6.01 for Windows; GraphPad Software, Inc.) statistical software was applied for statistical analysis. Student’s t-test or one-way ANOVA were used for comparison differences between groups. *P* < .05 was considered statistically significant.

## Results

### MSC-AS1 expression was reduced in ovarian cancer tissues and cell lines

**Fig. 1:**
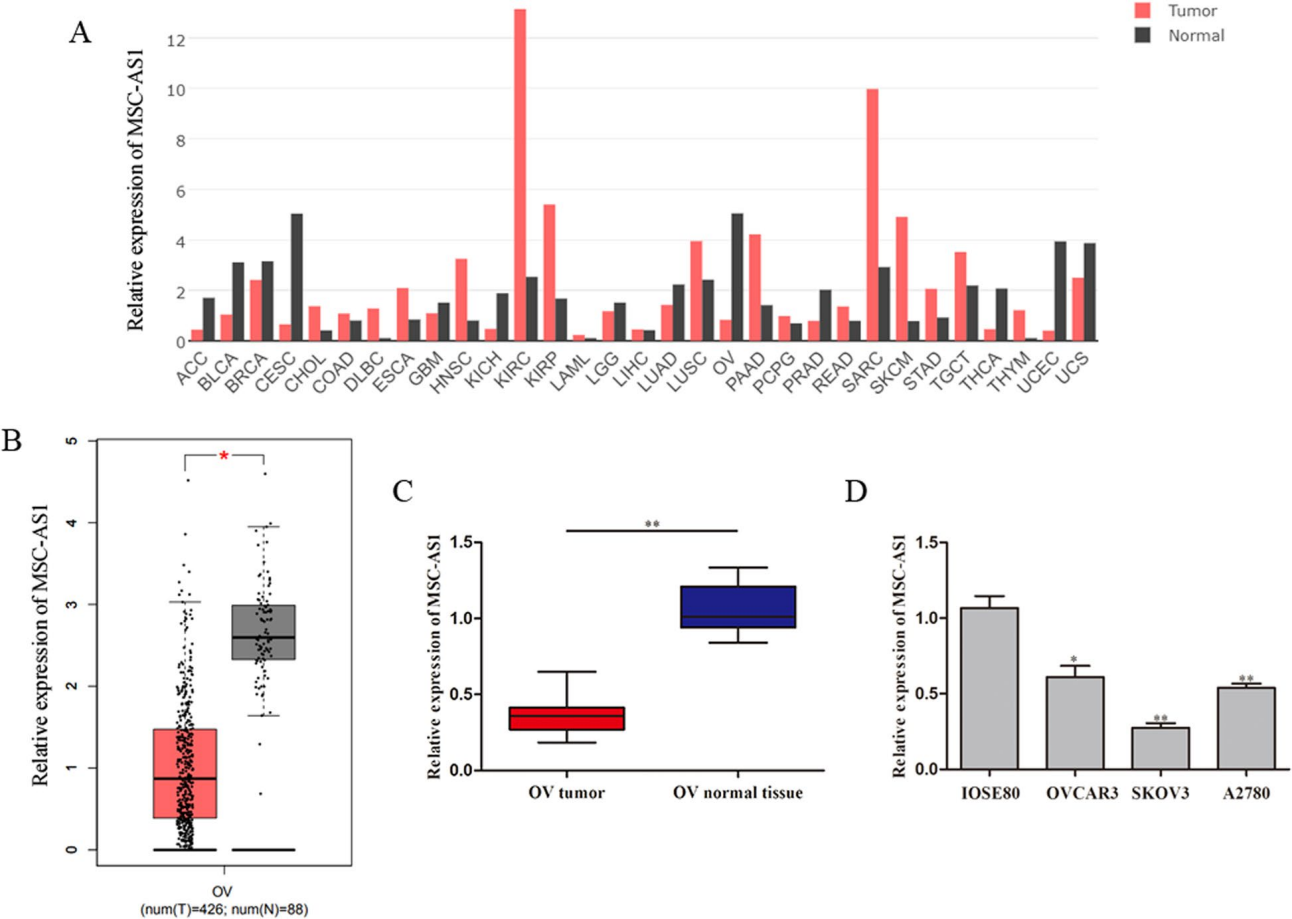
MSC-AS1 expression was reduced in ovarian cancer tissues and cell lines. **A**, **B** The expression of MSC-AS1 in ovarian cancer tissues and adjacent tissues was predicted using bioinformatics analysis based on GEPIA database. **C**, **D** qRT-PCR was used to detect the expression level of MSC-AS1 in ovarian cancer tissues (n=20) and cell lines. ^**^P<0.01 *vs*. Normal tissue or IOSE80

After browsing the Gene Expression Profiling Interactive Analysis (GEPIA) database (http://gepia.cancer-pku.cn/help.html) website [22], we discovered that MSC-AS1 expression in ovarian cancer tissues (n=20) was remarkably lower than that in normal tissues, as exhibited in Fig. 1A, B. Consistently, qRT-PCR assay showed that the expression level of MSC-AS1 was markedly lower in ovarian cancer tissues comparison with that in adjacent non-tumor tissues (Fig. 1C). Subsequently, the level of MSC-AS1 in ovarian cancer cell lines was examined. Similarly, MSC-AS1 was lowly expressed in three ovarian cancer cell lines (OVCAR3, SKOV3 and A2780) compared with that in human IOSE80 cells (Fig. 1D).

### MSC-AS1 over-expression inhibits proliferation of ovarian cancer cells

**Fig. 2:**
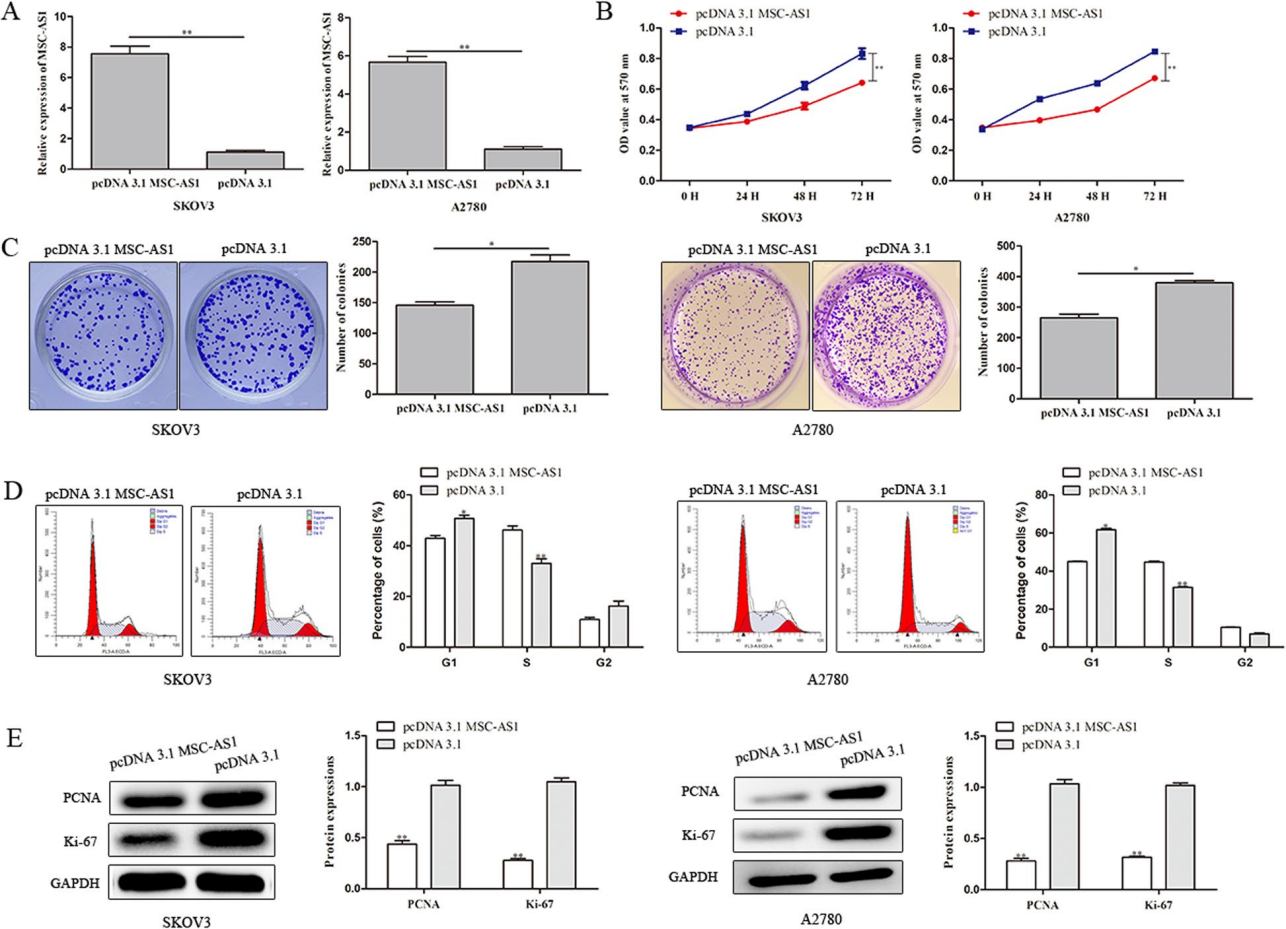
MSC-AS1 over-expression promotes proliferation of ovarian cancer cells. **A** The upregulation efficacies of MSC-AS1 in SKOV3 and A2780 cells were validated through qRT-PCR. **B**, **C** The impact of MSC-AS1 on cell viability and proliferation was explored by MTT and colony formation analyses. **D** Flow cytometry analysis was performed to explore the effects of MSC-AS1 on the cell cycle. **E** The protein expression levels of PCNA and Ki-67 were analyzed using western blot assay. ^*^P<0.05, ^**^P<0.01 *vs*. pcDNA 3.1

To probe the function of MSC-AS1 in ovarian cancer, overexpression plasmids were transfected into SKOV3 and A2780 cells to upregulate MSC-AS1 expression. The efficiency of transfection was confirmed by qRT-PCR (Fig. 2A). The MTT assay unveiled that MSC-AS1 overexpression markedly suppressed the viability of SKOV3 and A2780 cells (Fig. 2B). Consistently, the colony formation analysis revealed that pcDNA 3.1 MSC-AS1 significantly inhibited the proliferation of SKOV3 cells compared with pcDNA 3.1 group (Fig. 2C). In addition, flow cytometry and western blot analysis was performed to explore the effects of MSC-AS1 on the cell cycle. As shown in Fig. 2D, a significantly greater proportion of S phase cells were observed in SKOV3 and A2780 cells when MSC-AS1 was overexpression compared with pcDNA 3.1 group. On the basis of the protective function described above, several cellular molecules, including Ki-67 and proliferating cell nuclear antigen (PCNA), were also detected in this study. PCNA is a nuclear non-histone protein that stimulates DNA excision repair. Ki-67 is a classical marker of cellular proliferation. Western blot analysis indicated that MSC-AS1 over-expression decreased the expression levels of PCNA and Ki-67 compared to pcDNA 3.1 group (Fig. 2E).

### Overexpression of MSC-AS1 induces apoptosis of ovarian cancer cells

**Fig. 3:**
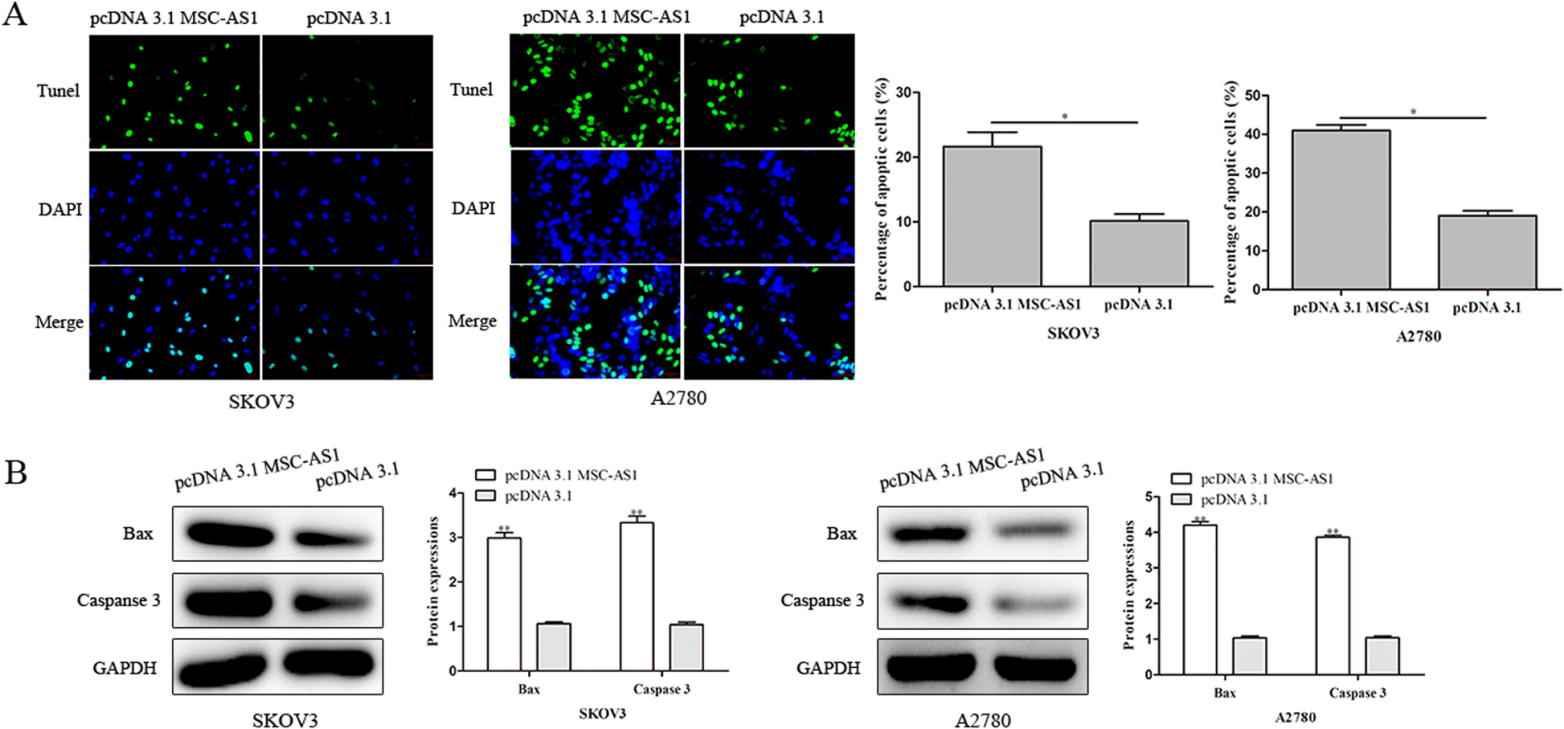
Impact of MSC-AS1 on ovarian cancer cell apoptosis. **A** The apoptosis property of SKOV3 and A2780 cells was investigated by tunel assay. **B** The apoptosis related protein expressions were investigated via western blot assay. ^*^P<0.05, ^**^P<0.01 *vs*. pcDNA 3.1

Flow cytometry was used detect the effect of MSC-AS1 over-expression on apoptosis of ovarian cancer cells. It was discovered from the results that pcDNA 3.1 MSC-AS1 induced of apoptosis rate in SKOV3 and A2780 cells compared with pcDNA 3.1 group (Fig. 3A). Furthermore, apoptosis related proteins were detected by western blot assay, and the results showed that MSC-AS1 over-expression resulted in an obvious rose in the expression levels of Bax and caspase-3 when transfected with pcDNA 3.1 MSC-AS1 in SKOV3 and A2780 cells (Fig. 3B).

### MiR-425-5p is a direct target of MSC-AS1

**Fig. 4:**
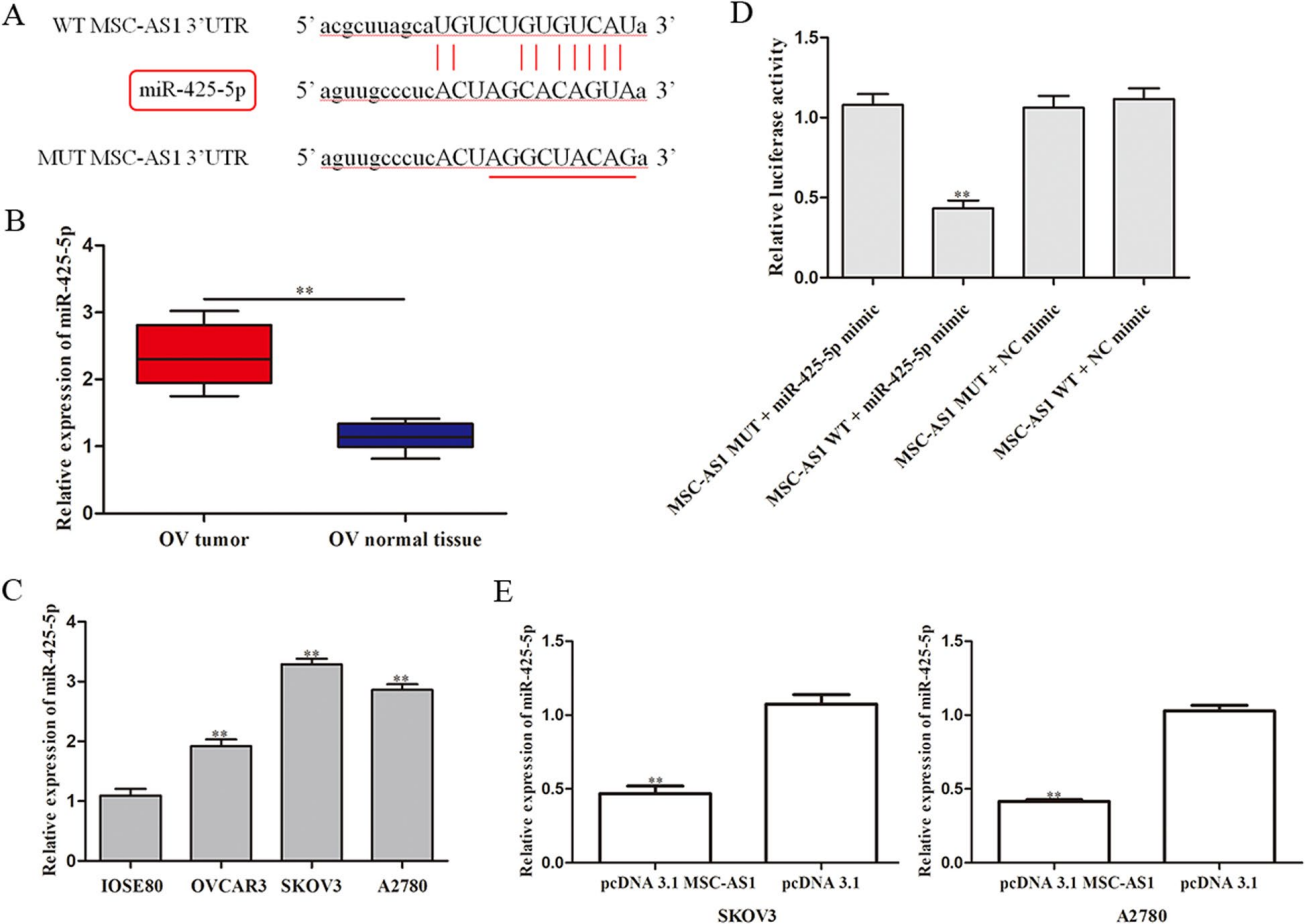
MiR-425-5p is a direct target of MSC-AS1. **A** The binding relationship between MSC-AS1 and miR-425-5p was assumed by StarBase datebase. **B** Level of miR-425-5p in ovarian cancer tissues and normal tissues was evaluated by qRT-PCR assay (n=20). **C** miR-425-5p expression was detected in ovarian cancer cell lines. **D** Luciferase reporter assay assessed the target of MSC-AS1 to miR-425-5p. **E** qRT-PCR analysis was used to measure the expression of miR-425-5p under transfection of pcDNA 3.1-MSC-AS1 or pcDNA 3.1. ^**^P<0.01 *vs*. Normal tissues, IOSE80, or pcDNA 3.1

To explore the mechanism underlying MSC-AS1 in ovarian cancer, StarBase database was utilized. As shown in Fig. 4A, 3’-UTR of miR-425-5p has been predicted as the candidate and contains the MSC-AS1 binding site. Next, the level of miR-425-5p in ovarian cancer tissues was examined and the result showed that the expression level of miR-425-5p was significantly up-regulated in ovarian cancer tissues comparing to normal tissues (Fig. 4B). Subsequently, the level of miR-425-5p was measured in OVCAR3, A2780, SKOV3 cells, and normal epithelial cells line (IOSE80). As presented in Fig. 4C, miR-425-5p expression in ovarian cancer cell lines was significantly higher compared to that in IOSE80 cells. Next, the target relationship between MSC-AS1 and miR-425-5p was verified through luciferase reporter assay. As expected, a significantly lower activity of the luciferase was observed in the combination of miR-425-5p mimic and MSC-AS1-WT but not MSC-AS1-MUT was weakened obviously after overexpression of miR-425-5p (Fig. 4D). qRT-PCR analysis was used to measure the expression of miR-425-5p (Fig. 4E). We demonstrated that MSC-AS1 over-expression decreased expression level of miR-425-5p in SKOV3 and A2780 cells.

### MSC-AS1 inhibits the ovarian cancer progression by targeting miR-425-5p

**Fig. 5:**
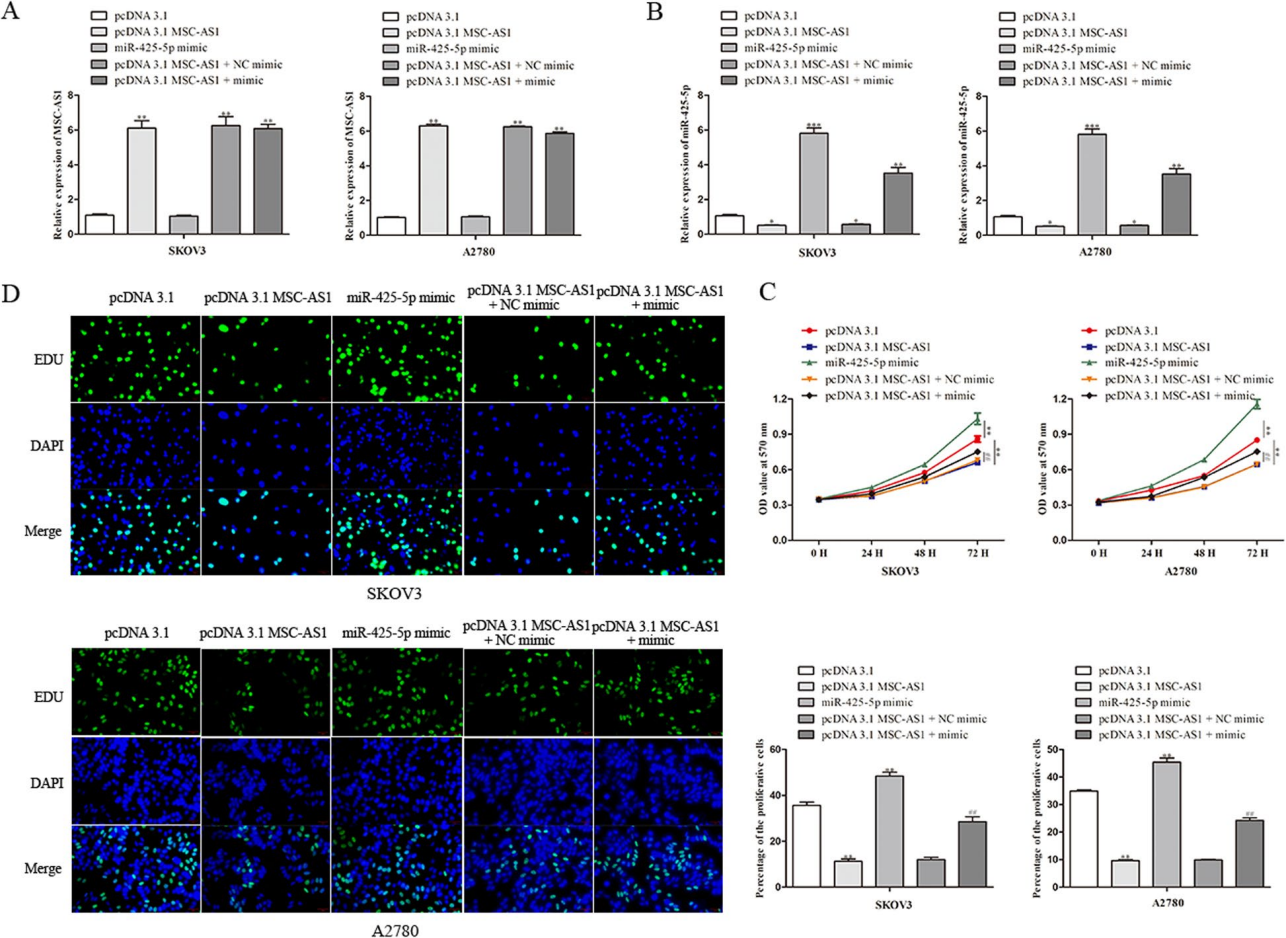
MSC-AS1 inhibits the ovarian cancer progression by targeting miR-425-5p. **A**, **B** Transfection efficiency was analyzed. **C**, **D** Cell viability and proliferation was measured by using MTT and EdU assays. ^**^P<0.01, ^***^P<0.001 *vs*. pcDNA 3.1. ^##^*P*<0.01 *vs*. pcDNA 3.1 MSC-AS1

Following the above experiments, we attempted to elucidate the mechanism underlying MSC-AS1. To perform rescue experiments, the miR-425-5p mimic was transfected into SKOV3 and A2780 cells with or without MSC-AS1 overexpression. Transfection efficiency is presented in Fig. 5A, B. The results presented that pcDNA 3.1-MSC-AS1 transfection decreased but miR-425-5p mimic transfection increased miR-425-5p expression, and that the level of miR-425-5p in cells co-transfected with pcDNA 3.1 MSC-AS1 and miR-425-5p mimic was almost similar to the original level of miR-425-5p. Then, the participation of miR-425-5p in MSC-AS1-mediated ovarian cancer cellular activity was explored. In MTT assay, it was observed that MSC-AS1 overexpression obviously boosted cell viability and miR-425-5p over-expression rescued the promotion impact of MSC-AS1-overexpression on cell viability (Fig. 5C). Same trends of cell proliferation were viewed in EdU experiments (Fig. 5D).

**Fig. 6:**
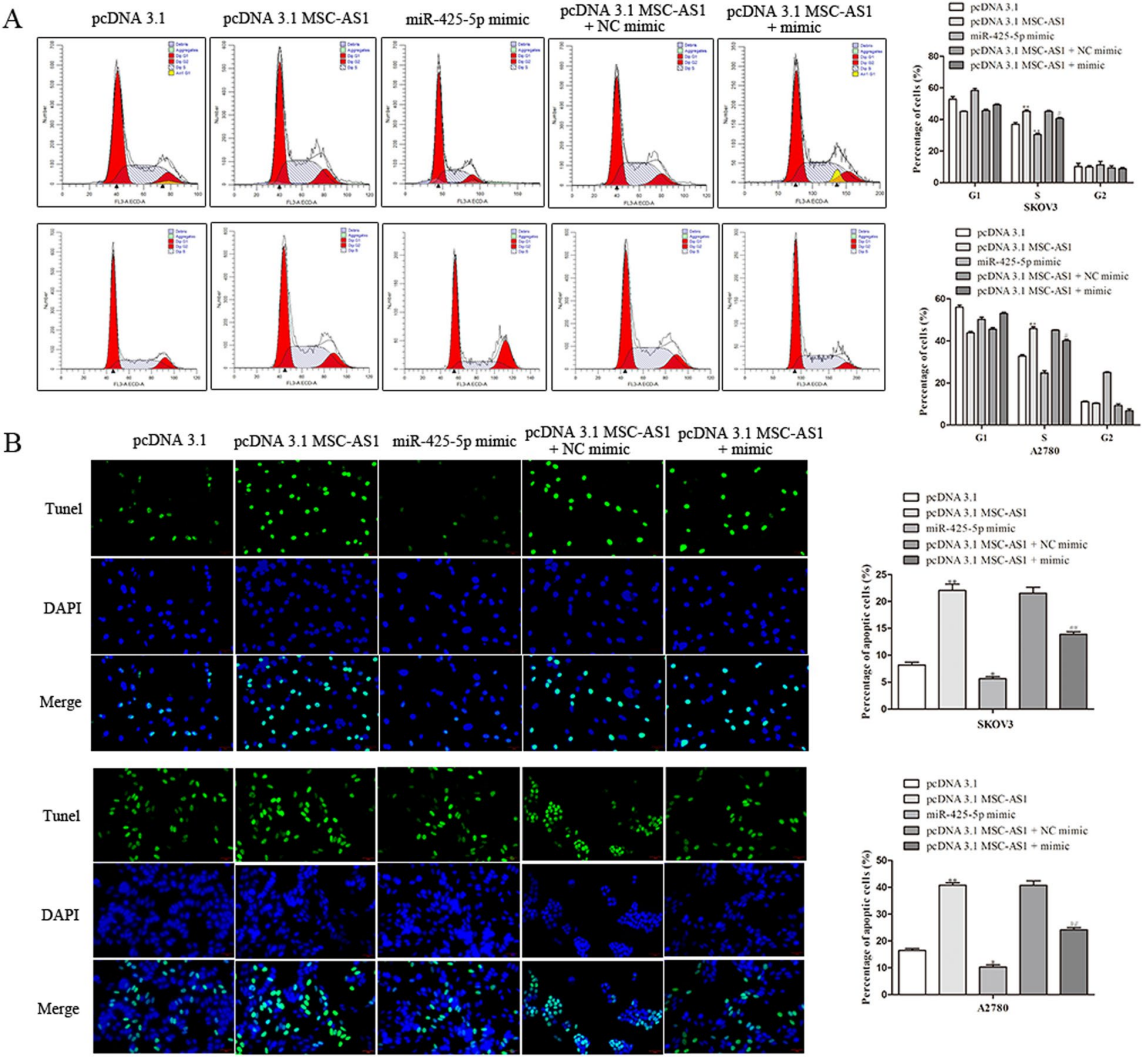
MSC-AS1 inhibits the ovarian cancer progression by targeting miR-425-5p. **A**, **B**. Determination of cell cycle and apoptosis by flow cytometry analysis. ^*^P<0.05, ^**^P<0.01 *vs*. pcDNA 3.1. ^#^*P*<0.05, ^##^*P*<0.01 *vs*. pcDNA 3.1 MSC-AS1

As shown in Fig. 6A, the flow cytometry experiment unveiled that MSC-AS1 promotion markedly increased the proportion of S phase cells were observed in SKOV3 and A2780 cells, while up-regulated miR-425-5p expression antagonized the impact of the pcDNA 3.1-MSC-AS1. Besides, flow cytometry results also revealed that after up-regulated MSC-AS1 expression, the apoptosis rate of SKOV3 and A2780 cells was significantly induced, while up-regulating miR-425-5p led to an opposite effect and up-regulating miR-425-5p was found to reverse the effects of MSC-AS1 overexpression on SKOV3 and A2780 cell apoptosis (Fig. 6B).

## Discussion

Although the rise of great advancements in therapeutic strategies, the survival rate of ovarian cancer patients remains very poor [23, 24]. Therefore, it is significant to further discover the molecular mechanisms related to ovarian cancer development and progression. The complex process of ovarian cancer includes gene expression, signal pathway and epigenetic changes. To study the abnormal expression of core genes [25], elucidate the molecular mechanism of ovarian cancer, and find potential biomarkers are of great significance for early diagnosis and prognosis, and help to find new treatment methods and improve the prognosis of patients with ovarian cancer.

In the past few decades, abnormal expression of lncRNAs has been widely found and plays a vital role in malignancies. Multiple studies have suggested that lncRNAs can participate in many pathological processes, including proliferation, apoptosis, invasion and differentiation [26–28]. With the development of high-throughput sequencing technology, TCGA and GEO have been established which as the major approach for selecting lncRNAs. We selected MSC-AS1 as a potential essential lncRNA for ovarian cancer progression and diagnosis and further studied the molecular mechanisms.

MSC-AS1 abnormal expressed in many tumors, such as pancreatic cancer, nasopharyngeal carcinoma, osteosarcoma and hepatocellular carcinoma [11, 29–31]. In the present study, MSC-AS1 expression was discovered to be down-regulated in ovarian cancer tissues and cell lines. Considering that the role of MSC-AS1 in ovarian cancer development and progression remained elusive, we intended to explore the precise role of MSC-AS1 in ovarian cancer. Subsequently, gain-of-function experiments validated that up-regulation of MSC-AS1 could significantly inhibit cell proliferation, clone formation, and induce apoptosis capacity of ovarian cancer cells. These data hinted that MSC-AS1 also played an anti-oncogenic role in ovarian cancer.

Growing evidence has demonstrated that the interaction between lncRNAs and miRNAs can form a complex regulatory network, which plays a vital role in the biological process of various malignancies [32, 33]. The possible mechanism of MSC-AS1 in ovarian cancer cells was also revealed. To validate the possible targets of MSC-AS1, miRDB (http://mirdb.org/) and StarBase 3.0 (http://starbase.sysu.edu.cn/) were utilized to predict the potentially target genes. 3’-UTR of miR-425-5p has been predicted as the candidate and contains the MSC-AS1 binding site. In addition, the dual luciferase reporter assay concluded that the interaction between MSC-AS1 and miR-425-5p is direct. A large number of studies have reported that miR-425-5p are abnormally expressed in a variety of malignant tumors, including breast cancer, lung cancer, colorectal cancer and gastric cancer [34–37], and that the altered expression of miR-425-5p is associated with the pathogenesis and progression of cancer. Subsequently, Rescue assays were also conducted to confirm that miR-425-5p over-expression could partially reverse the tumor suppressive function of MSC-AS1 overexpression, suggesting that MSC-AS1 can inhibit the development of ovarian cancer cells by down-regulating miR-425-5p expression.

In summary, we demonstrated that a new mechanism by which MSC-AS1 elevated miR-425-5p expression, which retard the progression of ovarian cancer, manifesting that MSC-AS1 serves as a promising target for the treatment of ovarian cancer patients.

## Data Availability

All data generated or analyzed using this study was included in this published article.

## Abbreviations

lncRNA MSC-AS1: Long non-coding RNA MSC-AS1
miR-425-5p: MicroRNA-425-5p
qRT-PCR: Quantitative real-time polymerase chain reaction
EdU: 5-Ethynyl-20-deoxyuridine
PI: Propidium iodide
TUNEL: Terminal deoxynucleotidyl transferase (TdT)-mediated dUTP nick end labeling
NC: Negative Control
